# Small Heat Shock Protein (*sHSP*) Gene Family from Sweet Pepper (*Capsicum annuum* L.) Fruits: Involvement in Ripening and Modulation by Nitric Oxide (NO)

**DOI:** 10.3390/plants12020389

**Published:** 2023-01-13

**Authors:** Salvador González-Gordo, José M. Palma, Francisco J. Corpas

**Affiliations:** Group of Antioxidants, Free Radicals and Nitric Oxide in Biotechnology, Food and Agriculture, Department of Stress, Development and Signaling in Plants, Estación Experimental del Zaidín, Spanish National Research Council (CSIC), C/Profesor Albareda 1, 18008 Granada, Spain

**Keywords:** fruit ripening, HSP20 family, nitric oxide, pepper, small heat shock proteins

## Abstract

Small heat shock proteins (sHSPs) are usually upregulated in plants under diverse environmental stresses. These proteins have been suggested to function as molecular chaperones to safeguard other proteins from stress-induced damage. The ripening of pepper (*Capsicum annuum* L.) fruit involves important phenotypic, physiological, and biochemical changes, which have associated endogenous physiological nitro-oxidative stress, but they can also be significantly affected by environmental conditions, such as temperature. Based on the available pepper genome, a total of 41 *sHSP* genes were identified in this work, and their distributions in the 12 pepper chromosomes were determined. Among these genes, only 19 *sHSP* genes were found in the transcriptome (RNA-Seq) of sweet pepper fruits reported previously. This study aims to analyze how these 19 *sHSP* genes present in the transcriptome of sweet pepper fruits are modulated during ripening and after treatment of fruits with nitric oxide (NO) gas. The time-course expression analysis of these genes during fruit ripening showed that 6 genes were upregulated; another 7 genes were downregulated, whereas 6 genes were not significantly affected. Furthermore, NO treatment triggered the upregulation of 7 *sHSP* genes and the downregulation of 3 *sHSP* genes, whereas 9 genes were unchanged. These data indicate the diversification of *sHSP* genes in pepper plants and, considering that *sHSPs* are important in stress tolerance, the observed changes in *sHSP* expression support that pepper fruit ripening has an associated process of physiological nitro-oxidative stress, such as it was previously proposed.

## 1. Introduction

Environmental heat stress has a negative impact on the growth, development, and productivity of crop plants and is considered a serious threat [1,2], where the metabolism of reactive oxygen and nitrogen species (ROS and RNS, respectively) is usually involved as a response mechanism [3,4,5,6,7,8,9]. Consequently, the plant-adaptive response to high temperatures is very important for plant development and also for food security worldwide. Under heat stress conditions, the heat-responsive genes, mainly those encoding heat shock proteins (*HSPs*), are switched on [10,11,12,13].

Plant HSPs are grouped into five main families based on the molecular weight and sequence homology, including *HSP100s*, *HSP90s*, *HSP70s*, *HSP60s*, and *HSP20s* [14]. Among all the five conserved families, *HSP20s*, a group of small HSPs (sHSPs) with molecular sizes ranging from 15 to 42 kDa, is the most prevalent and abundant family induced by heat stress in many higher plants [15,16,17,18]. These proteins function as molecular chaperones to safeguard other proteins from stress-induced damage. A common signature of the sHSPs is the alfa-crystallin domain (ACD) with around 90 amino acids that form a seven-stranded β-sandwich. This core is flanked by a variable N-terminal domain (NTD) with fewer than 85 amino acids and by a short C-terminal extension (CTE). The *sHSPs* are present in the cytosol and different organelles, including chloroplast, mitochondrion, endoplasmic reticulum, and peroxisome. Therefore, they bear the necessary and specific targeting signal either in the N- or C-terminal regions to lead them to diverse organelles. Usually, the majority of the *sHSPs* form multi-subunit oligomers in their native state [17,19,20,21].

Nitric oxide (NO) is a radical molecule that can exert regulatory functions of protein through posttranslational modifications, mainly *S*-nitrosation, tyrosine nitration, and metal nitrosylation [22,23,24,25], or through interaction with phytohormones, hydrogen peroxide, hydrogen sulfide, and melatonin, among others [26,27,28,29,30]. Furthermore, NO applied exogenously using either NO donors, gas or nanomaterials, has been shown to trigger beneficial effects on plants under physiological and stress conditions [31,32,33,34,35]. In the case of climacteric and non-climacteric fruits, NO has been shown to modulate the ripening process as well as to extend the postharvest storage [36,37].

Pepper (*Capsicum annuum* L.) plants have a significant economical relevance since their fruits constitute a group of horticultural products that are among the most consumed worldwide either fresh or processed. This relevance is related to their significant nutritional potentiality since pepper fruits are a source of vitamins (A, C, E, and B6), β-carotene, minerals, folic acid, and fiber [38,39]. Experimental data supporting the ripening of pepper fruits is associated to physiological nitro-oxidative stress [40,41], where the ROS metabolism is significantly regulated [42,43,44,45] and modulated by NO [46,47,48,49,50]. In a previous study, Guo and colleagues [51] analyzed the pepper *CaHSP20* genes family, focusing on their heat (40 °C, 2 h)-induced expression in various plant tissues, including root, stem, leaf, and flower. With this previous information, the present study focused on the analysis of the *sHSP* genes in the sweet pepper fruit using the RNAseq transcriptome obtained previously [46], and also on how the exogenous NO gas treatment could modulate their expression.

## 2. Results

### 2.1. sHSP Genes/Proteins from Pepper: Sequence, Structure, and Phylogenetic Analysis

**Figure 1:**
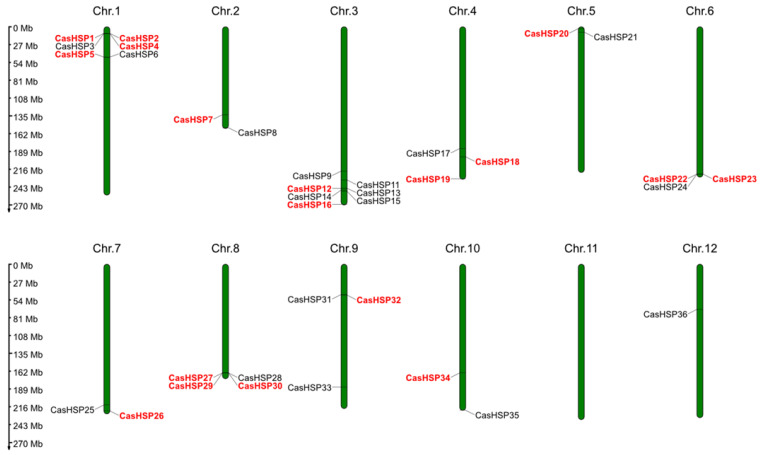
Chromosomal location of the *CasHSP* genes identified in pepper (*Capsicum annuum* L.) genome. The approximate locations of the 41 *CasHSP* genes among the 12 chromosomes are displayed in each chromosome (Chr.1–Chr.12). The distribution of the 19 *CasHSP* genes specifically detected in the sweet pepper fruit transcriptome is highlighted in red. Chr., chromosome.

*sHSPs* are a type of molecular chaperone with high diversity that are widespread in higher plants [17,52]. They are involved in protein homeostasis by binding proteins in non-native conformations, avoiding the irreversible aggregation of unfolded proteins. In this study, a total of 41 *sHSP* genes were identified and characterized in the pepper genome, which was named *CasHSP1* to *CasHSP41* based on their chromosomal location (Table 1). These 41 *CasHSP* genes were distributed across 12 pepper chromosomes (Figure 1). Thus, chromosome (Chr.) 3 contains 8 genes followed by Chr. 1, which has 7 genes, Chr. 8 with 4 genes, Chrs. 4 and 6 with 3 genes, Chrs. 2, 5 and 7 with 2 genes, and Chr. 12 with 1 gene. The only chromosome that does not contain any genes is Chr. 11. However, in the transcriptome obtained in sweet pepper fruits [46], only 19 *sHSP* genes, indicated in red in Table 1, were identified, suggesting that they are fruit-specific.

The identified *CasHSP* proteins, obtained from the nucleotide sequences, show a molecular weight ranging from 15.7 to 35.5 kDa and have a wide cellular distribution with 17 in the cytosol, 5 in the plastid, 1 in the peroxisome, 1 in the cytoskeleton, 1 in extracellular space, 1 in the vacuole, and 1 in the Golgi apparatus. Furthermore, there is dual localization of several *sHSPs* with 4 in plastid/mitochondrion, 3 in cytosol/nucleus, 2 in cytosol/mitochondrion, 4 in cytosol/Golgi, and 1 in Golgi/plastid.

**Figure 2:**
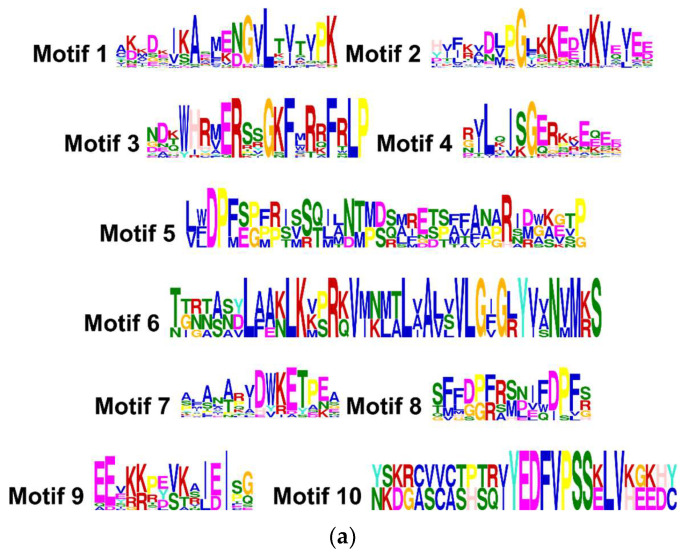

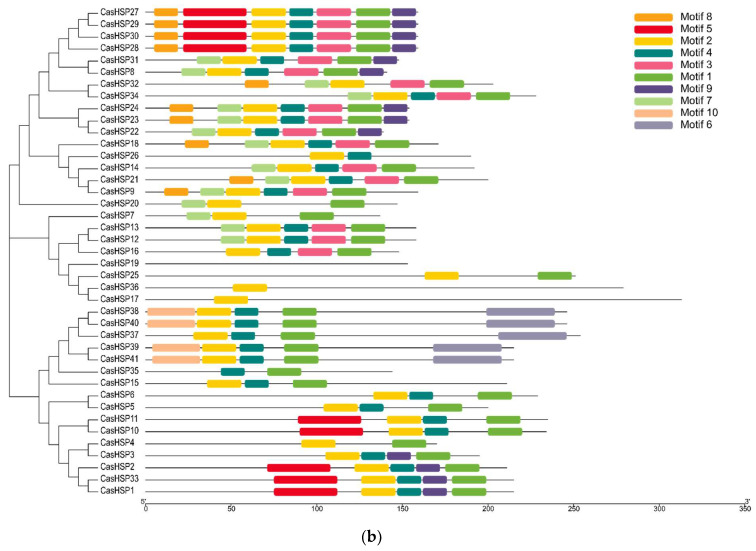
Identification and position of consensus amino acid motifs for pepper CasHSPs. (**a**) Amino acids motifs. Ten amino acid motifs with various widths were identified. The height of each amino acid symbol is proportional to the degree of conservation in the consensus sequences depicted in the ten motifs. (**b**) Distribution of conserved motifs. The distribution of conserved motifs, numbers 1–10, of the forty-one pepper sHSPs, are represented by boxes of different colors. Sequence logos of conserved motifs were created by MEME.

The analysis of the primary structure of the forty-one *sHSP* proteins revealed a high degree of identity with the families of *sHSPs* from other plant species, including Arabidopsis, tomato or potato, and allowed discriminating until ten amino acid motifs (Figure 2a). The distribution of these ten conserved motifs in the different *CasHSPs* from sweet pepper is represented in Figure 2b.

**Figure 3:**
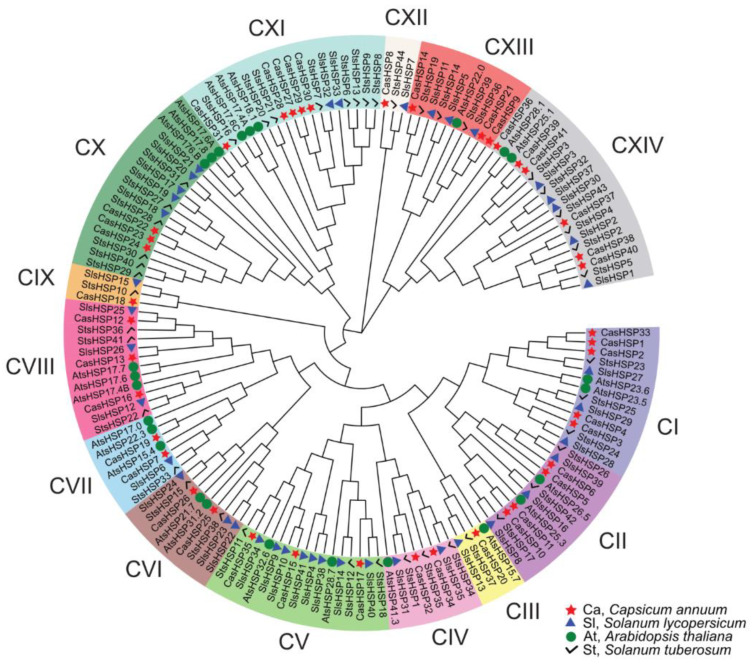
Phylogenetic analysis of plant small heat shock proteins (sHSPs). Identified CasHSP proteins in the fruit transcriptome are highlighted in red color. The scale bar represents the phylogenetic branch length. Different subgroups of sHSPs are depicted in different colors and designated as I to XIV. Species abbreviations: At (*Arabidopsis thaliana*), Ca (*Capsicum annuum*), Sl (*Solanum lycopersicum*), and St (*Solanum tuberosum*).

The phylogenetic comparative analysis among the sHSPs from four plant species including sweet pepper (*Capsicum annuum*), *Arabidopsis thaliana*, tomato (*Solanum lycopersicum*), and potato (*Solanum tuberosum*) allowed the identification of fourteen main sHSP groups, designated as I to XIV and depicted with different colors (Figure 3).

### 2.2. NO Gas Differentially Modulates sHSP Gene Expression during Pepper Fruit Ripening

**Figure 4:**
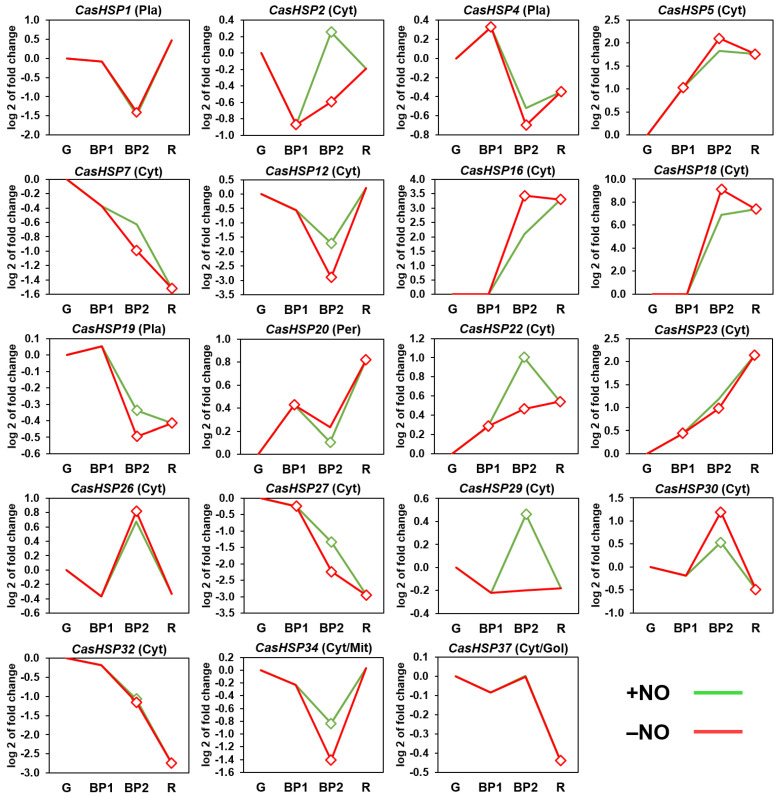
Time-course expression analysis of nineteen *CasHSP* genes (RNA-Seq) under natural ripening conditions and after exogenous NO gas treatment. Samples of sweet pepper fruits at different ripening stages correspond to immature green (G), breaking point 1 (BP1), breaking point 2 with (green line) and without (red line) NO treatment (BP2 + NO and BP2 − NO, respectively) and ripe red (R). Diamonds indicate statistically significant changes in expression levels (*p* < 0.05) in comparison to immature green (G). Cyt, cytosol. Mit, mitochondrion. Nuc, nucleus. Pla, plastid. Pex, peroxisome.

Based on previous analyses of sweet pepper fruits using the experimental design illustrated in Figure S1, three developmental steps were established: green immature (G), breaking point (BP1), and red ripe (R). Likewise, for the exposition to exogenous NO gas, two additional groups were established: fruits treated with 5 ppm NO for 1 h (BP2 + NO) and a parallel group that was not treated with NO (BP2 − NO), which was used as the control. Thus, the expression of the nineteen *sHSP* genes identified in the sweet pepper fruits was analyzed during ripening as well as the possible effect of NO gas treatment in comparison to untreated fruits. Figure 4 shows the time-course expression analysis of these nineteen *sHSP* genes by RNA-Seq at different stages of pepper fruit ripening. During ripening from green (G) to red (R) stages, it was possible to distinguish three groups of genes whose expression was increased (*CasHSP5*, *CasHSP16*, *CasHSP18*, *CasHSP20*, *CasHSP22*, and *CasHSP23*), decreased (*CasHSP4*, *CasHSP7*, *CasHSP19*; *sHSP27*, *CasHSP30*, *CasHSP32*, and *CasHSP37*) or not significantly affected (*CasHSP1*, *Ca*s*HSP*2, *sHSP12*, *CasHSP26*, *CasHSP29*, and *sHSP34*). 

On the other hand, independently of the modulation during ripening, under NO gas treatment at the BP2 stage, some of these genes were also either positive (*CasHSP2, CasHSP7*, *CasHSP12*, *CasHSP22*, *CasHSP27*, *CasHSP29*, and *Ca*s*HSP34*), negatively (*Ca*s*HSP16*, *Ca*s*HSP18*, and *CasHSP30*) or not significantly affected (*sHSP1*, *sHSP4*, *sHSP5*, *CasHSP19*, *CasHSP20*, *CasHSP23*, *CasHSP26*, *CasHSP32*, and *Ca*s*HSP37*) by NO treatment. Overall, a total of 10 *CasHSP* genes were modulated by NO.

## 3. Discussion

### 3.1. Pepper sHSP Genes Are Modulated during Ripening

Fruit ripening is a complex physiological process that involves high coordination among the different metabolic pathways that take place in the different subcellular compartments. Pepper is a non-climacteric fruit, which means that its ripening is independent of the phytohormone ethylene. Previous studies in pepper fruits have shown that the ripening implies significant changes at the transcriptome, proteome, and metabolic levels, and particularly there is a modulation of the metabolism of ROS and RNS [43,47,48,49,50] in a process that can be defined as a “physiological nitro-oxidative stress”, which has been also defined in the animal system as “oxidative eustress [53,54]. 

In a previous report, a search in the Pepper Genome Database (PGD, http://peppergenome.snu.ac.kr/ (accessed on 28 May 2022), CM334, Zunla-1, a variety which produced hot fruit) allowed to identify 36 genes of the *CaHSP20* family. The expression patterns of these genes were evaluated in four different tissues (root, stem, leaf, and flower) from two cultivars called B6 (thermo-sensitive) and R94 (thermo-tolerant) under heat stress. Among these genes, only 7 genes (*CaHsp16.4*, *18.2a*, *18.7*, *21.2*, *22.0*, *25.8*, and *25.9*) showed higher expression levels in both lines B6 and R94 under heat stress, and the rest of the genes showed what the author called “hysteretic expression” [51]; however, fruits were not analyzed in that work. In the present study, using our transcriptome from California-type sweet pepper fruits, cultivar Melchor, we analyzed the *sHSP* gene family because, to our knowledge, the information on sHSPs functions during fruit ripening, for either climacteric or non-climacteric fruits, are very limited. In our fruit system, we found 19 *sHSP* genes, which are 17 fewer than those found in pepper plants of the cultivar Zunla-1 [51]. This difference in the number of genes is notable and could be explained by the fact that our Melchor cultivar corresponds to California-type sweet peppers, while Zunla-1 produces hot peppers. In addition, our transcriptome derives exclusively from the fruits, which suggests that these 19 genes could be more specific to this plant organ. 

In tomato (*Solanum lycopersicum* cv Heinz 1706) plants, 33 *sHSP* genes have been reported and are mainly related to heat stress response and ripening [55]. In the case of tomato fruit which is a climacteric fruit, it was found that *HSP17.7* and sugar can interact to regulate the development of tomato fruit and affect its quality [56]. More recently, it was shown that ethylene and RIPENING INHIBITOR can regulate gene expression of the tomato *HSP17.7A* and *HSP17.7B*, which usually are upregulated during the ripening transition of mature green to breaker stage [57]. Another study of the sHSPs during ripening and storage at a low temperature in two tomato cultivars (Micro-Tom and Minitomato) and storage at a low temperature showed a differential response, where sHSPs were induced in Micro-Tom but not in Minitomato fruits. Thus, *sHSP17.4-CII* and *sHSP23.8-M* transcripts were strongly accumulated in Micro-Tom fruit, suggesting that these sHSPs could be involved in the mechanism of chilling tolerance in the Micro-Tom cultivar [58]. Moreover, early reports showed that the tomato sHSP21 participates in the protection of photosystem II against oxidative stress as well as promoting the color changes during fruit ripening through the carotene accumulation [59]. Moreover, an accumulation of sHSP during the chromoplast development during tomato ripening was reported [60].

On the other hand, using two pepper cultivars with different sensitivities to temperature, it was observed that the thermo-tolerant cultivar had a higher *CaHSP16.4* expression. Likewise, under combined stress of high temperature and drought, this cultivar had higher levels of antioxidant systems. Thus, the authors proposed that the higher expression of *CaHSP16.4* favors the scavenger capacity against ROS [61].

Similarly, in pepper plants, it has been shown that the *CaHsp25.9* expression increases under heat, salt, and drought stress, which confers tolerance since it was accompanied by a decrease in the accumulation of ROS, an increase of the activity of antioxidant enzymes, and upregulation in the expression of stress-related genes [62]. It should be mentioned that neither *CaHsp16.4* nor *CaHsp25.9* (named *CasHSP8* and *CasHSP6*, respectively, in Table 1) has been identified in pepper fruits. More recently, the expression analysis of *CaHSP18.1a* (named *CasHPS29* in Table 1 and expressed in fruit) in two pepper lines with different sensitivities to temperature (the thermo-sensitive B6 line and thermo-tolerant R9 line) showed that *CaHSP18.1a* is induced under heat stress, salt, and drought stress in both lines. Furthermore, the overexpression of *CaHSP18.1a* in Arabidopsis evidenced that this gene provides an increased resistance under these stresses since this transgenic Arabidopsis plants had also higher activity of some antioxidant enzymes, including catalase, SOD, and ascorbate peroxidase [63].

### 3.2. NO Modulates the Expression of Nine sHSP Genes during Pepper Ripening

NO is a well-recognized signal molecule that mediated many physiological processes including seed and pollen germination, root development, plant growth, stomatal movement, leaf senescence, flowering, and fruit ripening, and it is also involved in the mechanism of response against multiple adverse abiotic and biotic conditions [64,65,66]. Furthermore, new biotechnological approaches have been started to evaluate its potential application in crops using either NO donors or NO-releasing nanomaterial. In fact, postharvest storage has become one of the most attractive sectors to preserve horticultural products in good conditions and also to avoid losses due to pathogen attacks since NO can activate enzymatic and non-enzymatic antioxidant systems to palliate these negative outcomes [67,68,69].

The effect of exogenous NO gas on the pull of *CasHSP* genes during sweet pepper fruit ripening is, to our knowledge, unknown, and the provided data indicate that among the 19 identified *sHSP* genes, a total of 7 genes were upregulated and 3 were downregulated. It implies that, in a certain way, NO seems to restrict the effects triggered by the ripening process since a lower number of genes are influenced due to the treatment with the NO gas. In a previous study using the model plant *Arabidopsis thaliana*, the exogenous application of nitro-linolenic acid (NO*_2_*-Ln) which is a NO donor [6] triggered the induction of different families of HSPs including members of *HSP40*, *HSP60*, *HSP70*, and *HSP90* but most of them corresponded to sHSPs such as *Hsp17.6II* (At5g12020) or *Hsp17.6A* (At5g12030) [70]. Our data show that in pepper fruit the *CasHSP12* was positively regulated by NO. Consequently, our data provide additional evidence of the correlation between sHSPs and the signaling molecule NO which opens new questions to identify the mechanisms involved in this process. However, also, the knowledge of why and how the differential expression of these CasHSPs is orchestrated during ripening and by the NO treatment is a promising avenue that deserves to be investigated in the future.

In this sense, a more specific analysis, for example of the peroxisomal *CasHSP20* which encodes for an sHSP of 16.3 kDa provides some additional evidence about its potential function. In Arabidopsis, it was shown that two sHSPs are present in the peroxisomal matrix, specifically, AtsHSP15.7 (At5g37670) and AtsHSP31.2 (At1g06460), that are targeted by a functional peroxisomal targeting signal (PTS1) [SKL>] and a functional PTS2 (RLX_5_HF), respectively [71]. More recently, in this plant species, it has also been shown the presence of an additional sHSP, exactly the AtsHSP17.6 (At5g12020) which possesses unusual non-canonical PTS1, QKL, 16 amino acids upstream from the C-terminus and functions as a chaperone of the catalase, the main antioxidant enzyme present in peroxisomes which catalyzes the decomposition of H_2_O_2_ [72]. Thus, its overexpression confers higher catalase activity and tolerance to abiotic stresses [73]. In this sense, it should be mentioned that catalase of pepper fruit is negatively modulated by two NO-derived posttranslational modifications, S-nitrosation, and tyrosine nitration [50,72]; consequently, it could be suggested that the *CaHSP20* (16.3 kDa) may exert this function in pepper peroxisomes whose gene is significantly upregulated during ripening.

## 4. Materials and Methods

### 4.1. Identification of the sHSP Family Members

The set of pepper (*Capsicum annuum*, assembly UCD10Xv1.1) protein sequences was downloaded from the NCBI database (BioProject ID PRJNA376668). To identify the different sHSP candidates, we employed the HMMER3 software [74], which is based on the hidden Markov model (HMM) method. For this purpose, we obtained the alpha-crystallin/HSP20 domain (PF00011) from the Pfam database [75], which was used to search homologous sequences in the pepper proteome. Then, the presence of the conserved domain was confirmed using the SMART tool [76]. Proteins whose molecular weight was outside the range of 15–42 kDa were also rejected. A similar procedure was followed for the identification of sHSP members in *Arabidopsis thaliana* (assembly TAIR10.1), tomato (*Solanum lycopersicum*, assembly SL3.1), and potato (*Solanum tuberosum*, assembly SolTub_3.0).

### 4.2. Chromosomal Location, Phylogenetic and Conserved Motif Analyses of sHSP Sequences

Information about the chromosomal location of the identified CasHSPs was withdrawn from the NCBI database. The corresponding genetic map was constructed using the MG2C_v2.1 tool [77]. 

The sHSPs identified in sweet pepper, tomato, potato, and Arabidopsis were used to construct a phylogenetic tree. The alignment of sHSPs was performed using the MUSCLE method [78]. Then, the aligned sequences were subjected to MEGA11 [79] to perform an unrooted maximum likelihood phylogenetic tree with default parameters. Finally, the resulting phylogenetic tree was modified using the online tool Evolview_v3 [80].

Conserved motifs of CasHSPs were discovered using the MEME tool [81] and visualized using TBtools software [82]. The protein localization based on their amino acid sequences was predicted using WoLF PSORT [83].

### 4.3. Plant Material and Exogenous Nitric Oxide (NO) Gas Treatment

California-type sweet pepper cultivar Melchor fruits were collected from plants grown in plastic-covered greenhouses (Zeraim Iberica/Syngenta Seeds, Ltd., Roquetas de Mar/El Ejido, Almería, Spain). Fruits without any external damage were selected at three developmental stages: green immature (G), breaking point (BP1), and red ripe (R). Once harvested, these selected fruits were placed in black plastic bags and transported to the laboratory at room temperature, washed with distilled water, and kept for 24 h at a low temperature (about 7 °C ± 1 °C). For the analysis of the exogenous NO gas treatment, we set two additional groups: treated fruits with 5 ppm NO for 1 h (BP2 + NO) and another group that was not treated with NO (BP2 − NO) [37,81]. After 3 days, all fruits were chopped into small cubes (5 mm/edge), frozen under liquid nitrogen, and stored at 80 °C until use. Supplementary Figure S1 shows a representative picture of the experimental design followed in this study with the representative phenotypes of sweet pepper fruits at different ripening stages and subjected to NO treatment [46].

### 4.4. Library Preparation and RNA-Sequencing

All procedures were performed as previously described [46] with minor modifications. Briefly, libraries were prepared using an Illumina protocol and were sequenced on an Illumina NextSeq550 platform using 2 × 75 bp paired-end reads. These reads were pre-processed to remove low-quality sequences. Useful reads were mapped against the set of transcripts available for *Capsicum annuum* species in the NCBI database (assembly UCD10Xv1.1) using Bowtie2 [84]. Transcript counts were obtained using Samtools [85]. Differential expression analyses were done using DEgenes-Hunter [86]. This R pipeline examined the relative change in expression between the different samples using different algorithms (EdgeR, DESeq2, Limma, and NOISeq) which apply their own normalizations and statistical tests to validate the whole experiment. On the other hand, an analysis of the time course of *CasHSP* gene expression was performed considering as reference the expression levels found in green fruits (G). Raw data are accessible at the Sequence Read Archive (SRA) repository under the accession PRJNA668052. This reference pepper fruit transcriptome and differentially expressed (DE) genes among the analyzed ripening stages and the NO treatment involved the analysis of 24 biological replicates corresponding to 5 replicates of each stage, except for green fruits that involved 4 replicates.

## 5. Conclusions and Future Research Prospects

Nineteen *sHSP* genes were identified in sweet pepper fruits. The proteins encoded by these genes ranged from 16 to 28 kDa in size, and these sHSP proteins shared 10 motifs, but only motifs 6, 8, 9, and 10 were highly conserved. Pepper fruit sHSPs displayed a wide subcellular localization, including cytosol, plastids, mitochondria, nuclei, Golgi, and peroxisomes. Furthermore, 19 *sHSP* genes were differentially regulated by ripening, but the expression of 10 genes was also modulated by NO. Overall, it was found that sHSPs might be involved in the mechanism of fruit ripening and in the response to NO treatment, thus confirming the situation of stress undergone by fruits once this physiological process is triggered. In fact, the ripening of sweet pepper fruits is characterized to have a very active ROS and RNS metabolism, which is associated with a physiological nitro-oxidative stress. The anti-ripening effect of NO also corroborates that this signal molecule is possibly exerting a relevant role through this family of proteins to modulate a global response against the physiological stress conditions imposed by ripening. 

Future research should be aimed at identifying the molecular mechanisms by which NO can modulate specific *sHSP* gene expression, for example through NO-derived posttranslational modifications of specific transcription factors. However, it will be necessary to explore whether other signal molecules could also achieve these regulatory functions on sHSP. Among these signal molecules, it should be mentioned certain phytohormones (ethylene, abscisic acid, etc.), hydrogen sulfide (H_2_S), hydrogen peroxide (H_2_O_2_), or melatonin [25,27,28,36,87] whose functional network regulates numerous physiological processes, including the ripening of fruits.

## Figures and Tables

**Table 1:** Summary of the 41 small heat shock proteins (*sHSPs*) genes identified in the pepper (*Capsicum annuum* L.) genome and some of the properties related to the protein encoded for these genes and their subcellular localization. The nineteen *CasHSP* genes specifically detected in the sweet pepper fruit transcriptome are highlighted in red.

Gene Name	Gene ID	Chr.	Genomic Location	Protein ID	Length (aa)	kDa	Subcellular Localization
*CasHSP1*	107867615	1	9708054-9710773	XP_016569420.2	215	24.5	Plastid
*CasHSP2*	107867608	1	9725633-9727173	NP_001311883.1	211	24.1	Cytosol
*CasHSP3*	107852753	1	10229995-10231900	XP_016553288.2	195	21.5	Plastid
*CasHSP4*	107852746	1	10242027-10244276	XP_016553275.2	170	18.4	Plastid
*CasHSP5*	107845765	1	46048175-46056638	XP_047266663.1	200	17.7	Cytosol
*CasHSP6*	107874604	1	46076284-46077623	XP_016576855.1	229	25.9	Plastid/Mitochondrion
*CasHSP7*	107861257	2	133802215-133803014	XP_016562092.1	137	15.7	Cytosol
*CasHSP8*	107860709	2	152930539-152932661	XP_016561651.1	141	16.4	Cytosol
*CasHSP9*	107865071	3	219296047-219302039	XP_047264613.1	159	18.5	Cytosol/Nucleus
*CasHSP10*	107863044	3	232468924-232469818	XP_047263866.1	234	25.7	Plastid
*CasHSP11*	107863044	3	232468924-232469818	NP_001311776.1	235	25.7	Plastid
*CasHSP12*	107862598	3	245438867-245439343	XP_016563712.2	158	17.7	Cytosol
*CasHSP13*	107862597	3	245535719-245536195	XP_016563710.2	158	17.7	Cytosol
*CasHSP14*	107862438	3	248116668-248117246	XP_016563509.1	192	19.1	Extracellular
*CasHSP15*	107865962	3	249658853-249659681	XP_016567632.1	211	24.0	Cytosol
*CasHSP16*	107861648	3	270049897-270050678	XP_016562442.1	148	16.5	Cytosol
*CasHSP17*	107869601	4	185294212-185295359	XP_016571594.2	313	35.5	Cytosol
*CasHSP18*	107853496	4	197561845-197562964	XP_016553971.2	171	18.4	Cytosol
*CasHSP19*	107866765	4	231026649-231027604	XP_016568214.1	153	16.7	Plastid
*CasHSP20*	107870139	5	2638802-2640752	XP_016572055.1	147	16.3	Peroxisome
*CasHSP21*	107872316	5	7720799-7721401	XP_016574557.1	200	22.6	Vacuole
*CasHSP22*	107875508	6	223245724-223246143	XP_016577738.2	139	17.8	Cytosol
*CasHSP23*	107875506	6	223249290-223249754	XP_016577734.2	154	17.8	Cytosol
*CasHSP24*	107875505	6	223271811-223272275	XP_016577732.1	154	17.6	Cytosol
*CasHSP25*	107877347	7	213882206-213884807	XP_016579494.1	251	27.6	Plastid
*CasHSP26*	107856376	7	223234365-223235748	XP_016556884.1	190	21.7	Cytosol
*CasHSP27*	107840198	8	164788707-164789186	XP_016539463.1	159	18.2	Cytosol
*CasHSP28*	107879949	8	164801975-164802454	XP_016582357.1	159	18.2	Cytosol
*CasHSP29*	107840199	8	164826536-164827015	XP_016539464.1	159	18.1	Cytosol
*CasHSP30*	107840200	8	164831732-164832211	XP_016539465.1	159	18.1	Cytosol
*CasHSP31*	107842139	9	45981288-45981734	XP_016541392.1	148	17.2	Cytosol
*CasHSP32*	107842861	9	46468003-46469487	XP_016542384.1	203	23.8	Cytosol
*CasHSP33*	107841934	9	186338719-186339366	XP_016541270.2	215	24.3	Plastid/Mitochondrion
*CasHSP34*	107844800	10	164686556-164687242	XP_016544631.2	228	26.5	Cytosol/Mitochondrion
*CasHSP35*	107845199	10	221623125-221623644	XP_016544930.1	144	16.6	Cytosol/Golgi
*CasHSP36*	107850800	12	68905209-68906648	XP_016551048.1	279	31.6	Cytosol/Nucleus
*CasHSP37*	107848440	Unplaced	532-1187	XP_047258842.1	254	28.4	Cytosol/Golgi
*CasHSP38*	107855006	Unplaced	357733-359091	XP_016555471.1	246	26.9	Golgi
*CasHSP39*	107855027	Unplaced	665247-666808	XP_016555490.1	215	24.3	Cytosol/Golgi
*CasHSP40*	124890240	Unplaced	3954230-3955587	XP_047258018.1	246	26.8	Golgi/Plastid
*CasHSP41*	124890244	Unplaced	4261704-4263265	XP_047258024.1	215	24.3	Cytosol/Golgi

## Data Availability

Sequence Read Archive (SRA) data are available at the following link https://www.ncbi.nlm.nih.gov/sra/PRJNA668052 (accessed on 28 May 2020).

## Supplementary Materials

The following supporting information can be downloaded at: https://www.mdpi.com/article/10.3390/plants12020389/s1, Figure S1: Illustrative picture showing the experimental design used in this study with the representative phenotype of sweet pepper (*Capsicum annuum* L.) fruits at different stages and treatments.
