# Supplementary material for: Small Heat Shock Protein (sHSP) Gene Family from Sweet Pepper (Capsicum annuum L.) Fruits: Involvement in Ripening and Modulation by Nitric Oxide (NO)
Source: Plants (Basel). 2023 Jan 13;12(2):389. doi: 10.3390/plants12020389 (PMC9861568; doi:10.3390/plants12020389)
Supplement: Supplementary file 1 [file plants-12-00389-s001.zip › plants-2098288-supplementary.pdf]

**Supplementary Materials**

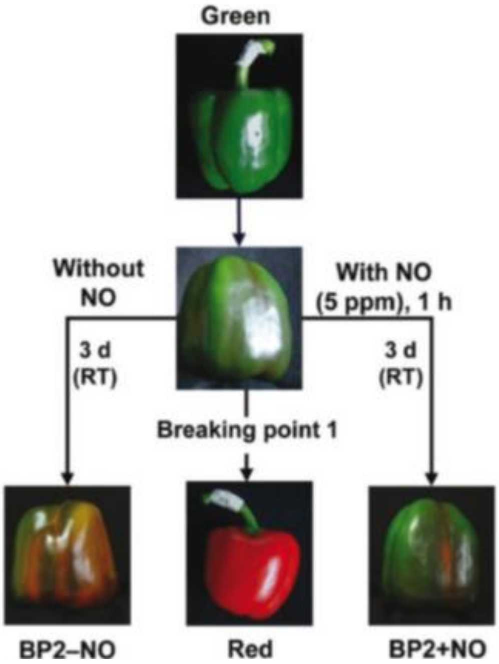


**Figure S1**. Illustrative picture showing the experimental design used in this study with the representative phenotype of sweet pepper (*Capsicum annuum* L.) fruits at different stages and treatments: immature green, breaking point 1 (BP1), breaking point 2 without NO treatment (BP2 – NO), breaking point 2 with NO treatment (BP2 + NO), and ripe red. Fruits were subjected to a NO-enriched atmosphere (5 ppm) in a hermetic box for 1 h and were then stored at room temperature (RT) for 3 days. Reproduced with permission from González-Gordo et al. [45].
